# The durability of flexible eddy current array (FECA) sensors in harsh service environments

**DOI:** 10.1038/s41598-021-89750-y

**Published:** 2021-05-14

**Authors:** Yujian Song, Tao Chen, Ronghong Cui, Yuting He, Xianghong Fan, Binlin Ma

**Affiliations:** Aeronautic Engineering College, Air Force Engineering University, 1 Baling Road, Xi’an, 710038 China

**Keywords:** Engineering, Materials science

## Abstract

Sensors for structural health monitoring (SHM) need to be permanently integrated on structures and withstand the harsh service environments, which has been a big challenge for the application of SHM in aircrafts. This paper focuses on the durability of flexible eddy current array (FECA) sensors in harsh service environments of aircrafts, including vibration environment and several typical exposed environments. First, a kind of FECA sensor is illustrated and its integration method is proposed. Moreover, in order to study the durability of the sensor in vibration environment, the modal analysis is performed by the finite element method. According to the simulation results, the durability experiment in vibration environment is carried out under the fourth order vibration mode, which makes the sensor suffer the harshest vibration loads. During the vibration experiment, output signals of the sensor keep stable and the sensor is well bonded to the structure, which shows the integrated sensor has high durability in vibration environment. Finally, the durability of integrated sensors is separately tested in three exposed environments, including salt fog corrosion environment, fluid immersion environment, as well as hygrothermal and ultraviolet-radiation environment. After these environmental exposure experiments, all sensors are well bonded to structures and can effectively monitor fatigue cracks, which shows great durability. Therefore, FECA sensors can survive in harsh service environments of aircrafts, which provides important support for the engineering applications of FECA sensors.

## Introduction

During the service of aircrafts, due to various loads and environmental conditions, the structural strength of metal structures will gradually decrease, and various damages such as fatigue and corrosion will occur, resulting in structural failure. Cracks are the most direct and ultimate damage mode of metal structures^1^. SHM technology can monitor important components of aircraft structures. It has been an important method to prevent and reduce hidden safety hazards and catastrophic accidents^2^. SHM technology for aircrafts uses sensors integrated on aircraft structures to obtain and process information to determine the health state of the aircraft structure^3,4^.

Current sensors for SHM include Piezoelectric transducers^5^, fiber optic sensors^6–8^, Comparative Vacuum Monitoring (CVM) sensors^9^, eddy current sensors^10,11^, acoustic emission sensors^12^, smart coating sensors^13,14^, ultrasonic sensors^15,16^ and so on. FECA sensors manufactured by the flexible printed circuit board technology have the ability to monitor the structure without direct contact, and they can be used to detect the damage of components with complex geometric shapes.

JENTEK Sensors^17,18^ developed a series of meandering winding magnetometer (MWM) array eddy current sensors for detecting micro damages of metal structures, and carried out much research in the quantitative monitoring of structural cracks. But they have not studied the influence of the service environment on the durability of sensors. Xie et al.^19^ developed a novel FECA sensor composed of 64 elements for NDI of aircraft engine blades, with an accuracy within ± 0.2 mm. Chen et al.^20,21^ improved the detection sensitivity of the sensor by optimizing the layout and excitation method of the planar eddy current sensor, and effectively suppressed the interference caused by the lift-off effect. Zhang et al.^22^ studied a FECA sensor for crack detection of curved structures, and established a method to eliminate the effect of lift-off effect. Sun et al.^23^ proposed an eddy current array sensing film adhered to bolts to quantitatively monitor the radial and axial crack growth of holes in aircraft bolted metal joints. Fan et al.^24^ proposed a new excitation coil arrangement method for the traditional rosette type flexible eddy current array sensor, which greatly improved the sensor's sensitivity to crack identification. Chen et al.^25–27^ proposed a method to improve the sensitivity of the FECA sensor, and solved the influence of the environmental temperature change on the output signal. It can be seen that current researches on sensors for SHM mainly focuses on the realization of the sensor function, signal processing methods and other basic problems. Researches related to service environments are only limited to environmental impacts on output signals of sensors, and the durability of sensors for SHM of aircrafts in harsh service environments has not been studied in detail.

However, aircraft structures work in harsh environments, including salt fog corrosion environment, fluid immersion environment, as well as hygrothermal and ultraviolet-radiation environment. These environments have adverse impacts on the durability and reliability of sensors, which may result in their failure or falling off from structural surface. And because of the long service life of structures, the durability of sensors is more difficult to meet the requirements. At the same time, when sensors are used on the structure, it is usually necessary to use an adhesive to stick on the surface of the structure. The adhesive tends to have a higher hardness after curing, which also easily causes the problem of insufficient durability. Thus, the durability of sensors in service environments of aircraft has been a big challenge for the application of SHM in aircrafts.

Therefore, a kind of FECA sensor is taken as the research object, and the durability of the sensor in harsh service environments of aircrafts is studied for the first time. Firstly, the composition of the sensor is analyzed, and the use of aviation sealant for integrating is proposed. Then, based on the results of finite element simulation analyses, a vibration environment durability experiment is carried out to study the durability and output signal stability of the sensor in vibration environment. Finally, the durability experiments in the exposed environments are carried out, including salt fog corrosion environment, fluid immersion environment, as well as hygrothermal and ultraviolet-radiation environment. Compared with other related researches, the innovation of this paper is to propose an integration method of the FECA sensor and study the durability of FECA sensors in vibration environment and several typical exposed environments for the first time, which provides important support for the engineering applications of FECA sensors.

## FECA sensor and integration method

### FECA Sensor

The FECA sensor is a kind of eddy current sensor which is manufactured by flexible printing circuit board technology and consists of multiple arrayed coils. FECA sensor can be used for quantitatively monitoring cracks of structures. FECA sensors are mainly divided into annular FECA sensors and rectangular FECA sensors according to the shape and function. Rectangular FECA sensors are mainly used for crack monitoring of curved structures and welded structures, and annular FECA sensors are mainly used for crack monitoring of bolt-jointed structures. In this paper, a kind of annular FECA sensor is selected as the research object to study the durability of FECA sensors in harsh service environments.

**Figure 1:**
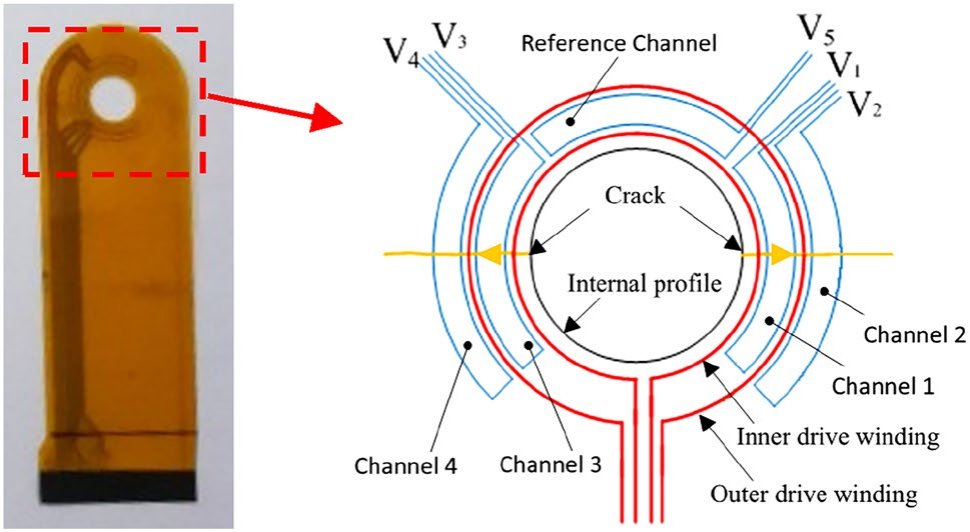
The annular FECA sensor.

As shown in Fig. 1, the sensor consists of two separate drive coils and four sense channels. The output signal of the sensor is defined as:

1 $$\Delta C = \frac{{C_{r} - C_{m} }}{{C_{r} }} \times 100\%$$

In the equation, ΔC is the characteristic signal, $C_{r}$ is the conductivity of reference channel, and $C_{m}$ is the conductivity of measure channel. Actually, the output signal ΔC represents the variation of the measure channel relative to the reference channel.

**Figure 2:**
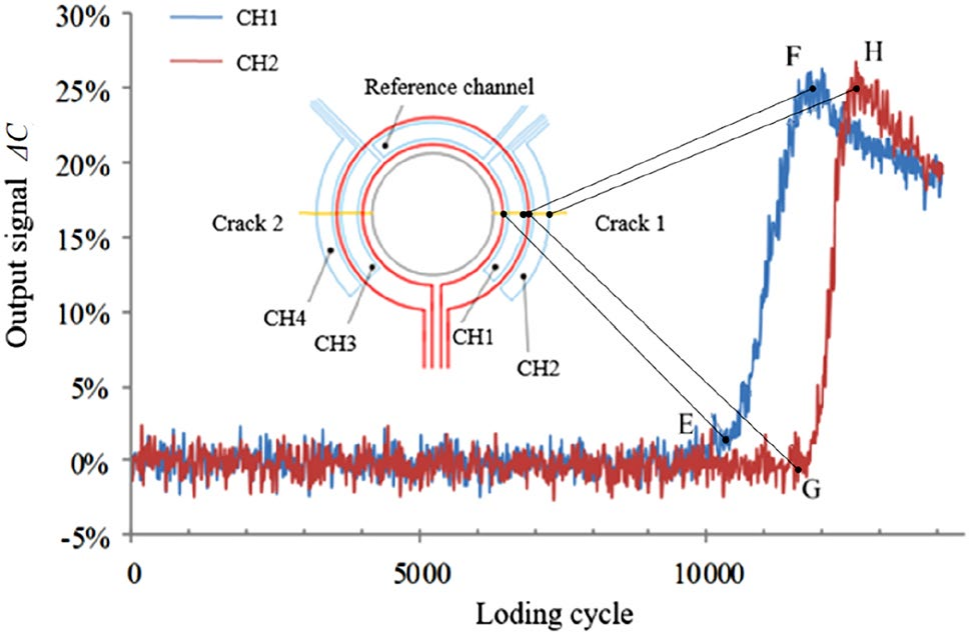
The relation between the crack length and output signals of the sensor.

As shown in in Fig. 2, when the output signal of the sense channel reaches a flection point (E, F, G, or H), the crack tip just arrives at corresponding coils, which can be used to quantitatively monitoring cracks according to the distance between adjacent coils.

### Sensor integration method

**Figure 3:**
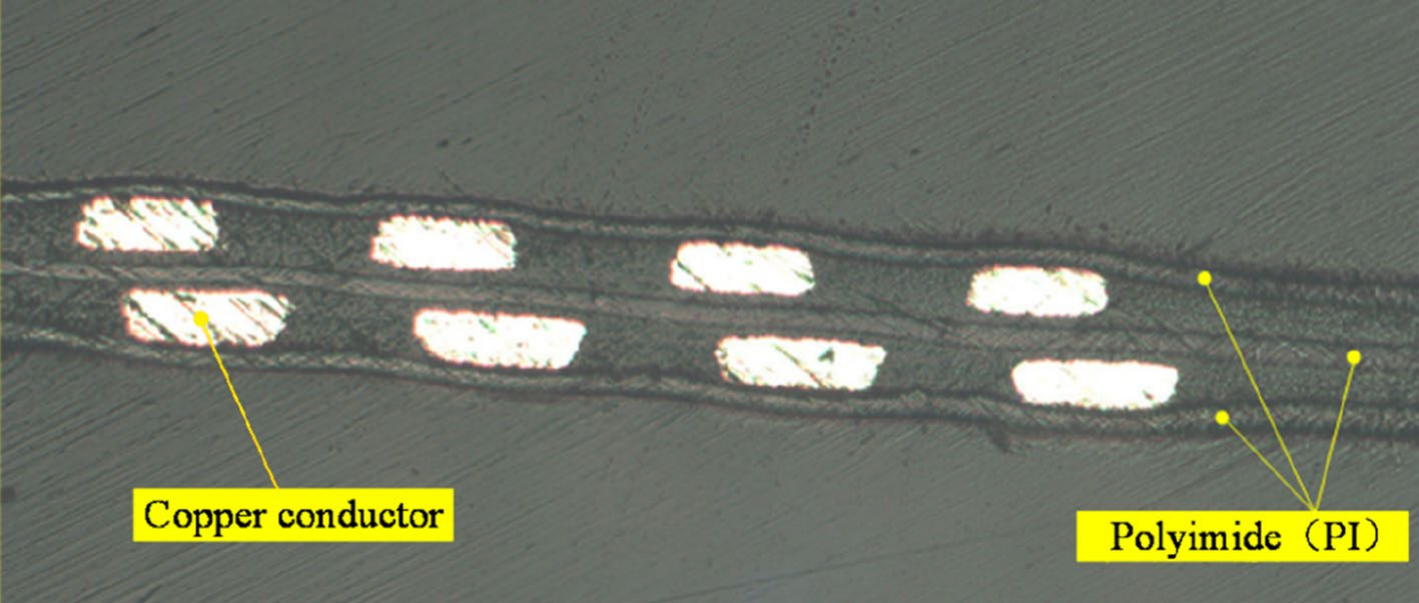
Fracture morphology of flexible eddy current array sensor observed under metallographic microscope.

FECA sensors are manufactured by flexible printing circuit board technology. The drive coils and the sense coils are distributed on both sides of the substrate layer, and the surface is covered with a protective film. In order to observe the actual structure of the sensor after manufacturing, a fractured sample of the sensor is prepared and observed under a metallographic microscope. The fracture morphology of flexible eddy current array sensor observed under metallographic microscope is shown in Fig. 3. It can be seen from the figure that the copper wire is attached to the intermediate polyimide (PI) substrate layer, and the outer surface are PI protective layers. PI has very good mechanical properties, abrasion resistance, heat resistance, corrosion resistance and aging resistance, and has high reliability even in various complex environments^28^. Therefore, FECA sensors manufactured by flexible printing circuit board technology have a certain durability.

**Figure 4:**
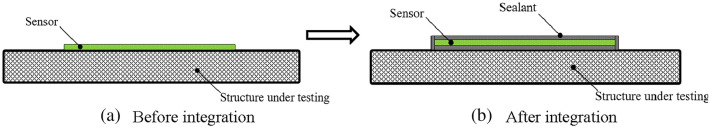
Schematic diagram of sensor integrated.

However, it is obvious that PI protective layers cannot guarantee the FECA sensor to work normally in harsh environment for a long time. Besides, when FECA sensors are used for structural crack monitoring, they need to be integrated with the structure. Therefore, it is necessary to take an effective method to integrate and protect the sensor, so as to realize the long-term normal operation of the sensor in the harsh environment. Some special aviation sealants can be used to seal the fuel tank and air system of aircrafts. The aviation sealant is an elastomer after curing, which can ensure that the sealant will not damage the sensor due to its own curing while protecting the sensor from the external environment. Moreover, the aviation sealant has the characteristics of oil resistance, solvent resistance, water resistance, aging resistance, corrosion resistance, and good mechanical properties and adhesive properties. Therefore, HM109 aviation sealants are used to integrate and protect the sensors in this paper. The integration principle of sensor is shown in Fig. 4, and the specific method steps are as follows:

1. Remove the dirt, dust and other sundries on the structure surface and dry it;
2. Mix the base paste and vulcanizing paste of the sealant according to a ratio of 10: 1 by mass;
3. Apply the prepared sealant evenly on the monitoring surface of the sensor, and smooth it with a smooth finish;
4. Stick the sensor on the surface of the structure under testing, and seal the outer surface of the sensor with a sealant;
5. Cure the Integrated sensors at room temperature for 24 h or at 70 °C for 2 h.

## The durability experiment in vibration environment

In the use of aircraft structure, airflow and engine working can cause structural vibration. The impact of vibration on the sensor may be manifested in two aspects: on the one hand, as sensors are adhered to the structure surface through sealant, the sealant and the sensor would vibrate with the structure at the same time, which may make the sealant debonding; on the other hand, the lift-off distance between the sensor and the structure is approximately 0.2 mm. It is the vibration environment that causes the lift-off distance changing, thereby greatly increasing the fluctuation amplitude of the output signal. Therefore, this section conducts the vibration experiment to verify the durability and reliability of the integrated sensor.

### Vibration modal analyses

**Figure 5:**
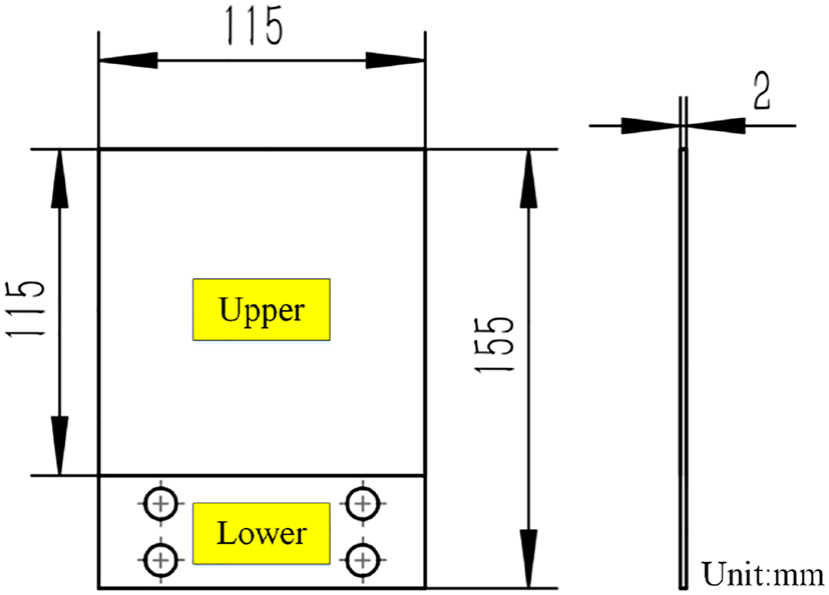
The vibration experiment specimen.

**Figure 6:**
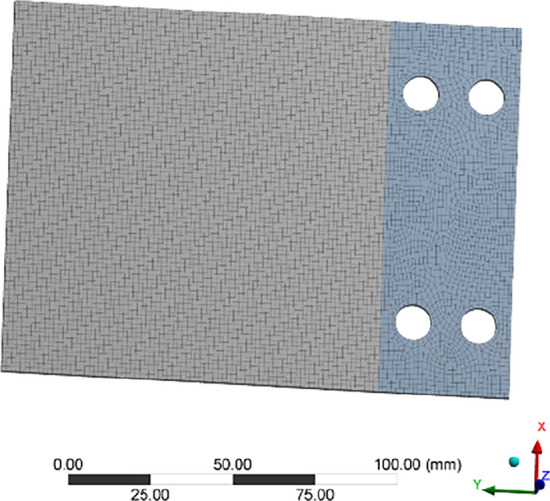
Finite element model of the specimen.

As is shown in Fig. 5, A 2024-T351 aluminum alloy specimen is designed in this paper to carry out the durability experiment in vibration environment. The upper part of the specimen is the vibration part and the lower part is the clamping area. As shown in Fig. 6, the boundary condition of the lower part (the blue part) is set as fixed support, and the modal analysis of the specimen is carried out.

**Figure 7:**
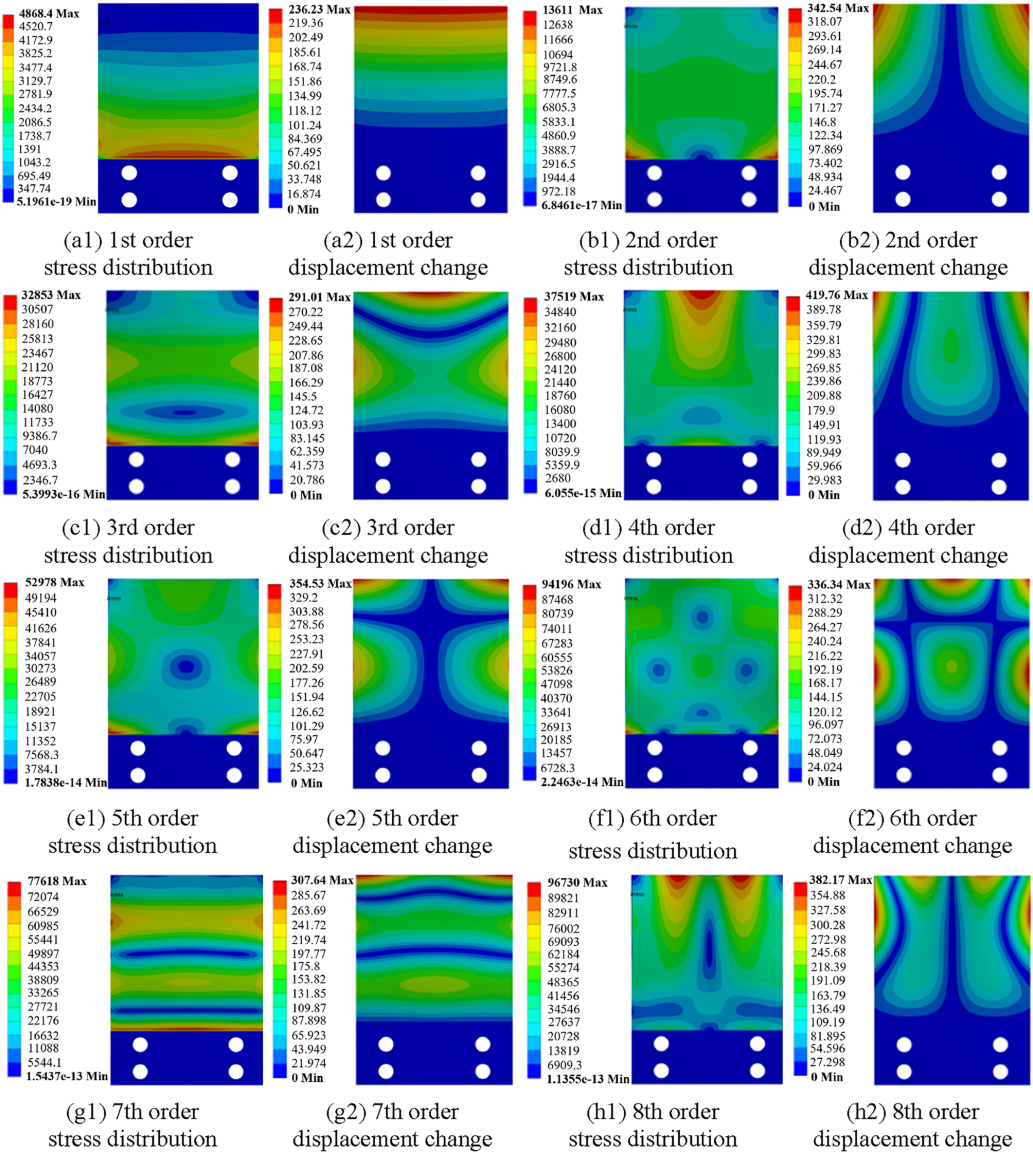
Stress distribution and displacement change of the specimen in each order mode (unit: MPa).

As shown in Fig. 7, the stress distribution and displacement changes of the first eight orders are obtained by the finite element modal analyses. Meanwhile, the resonance frequencies corresponding to each order are shown in Table 1. As illustrated in Fig. 7, the stress distribution and deformation of the specimen under different vibration modes are different. Except for the 4th and 8th modes, the stress concentration areas of the specimen are all near the clamping end, which is not suitable for installing FECA sensors. However, the resonance frequency of the 8th order mode is 2358.5 Hz, which is much higher than the optimal working frequency of the vibration equipment. Therefore, in order to effectively exert the efficiency of the vibration equipment, the fourth order vibration mode with a resonance frequency of 1005 Hz is selected for experiment.

**Table 1:** Resonance frequencies corresponding to each order mode of the specimen.

Modal order	1st	2nd	3rd	4th	5th	6th	7th	8th
Resonance frequency (Hz)	129.0	310.3	785.2	1005.0	1132.6	1978.2	2269.2	2358.5

**Figure 8:**
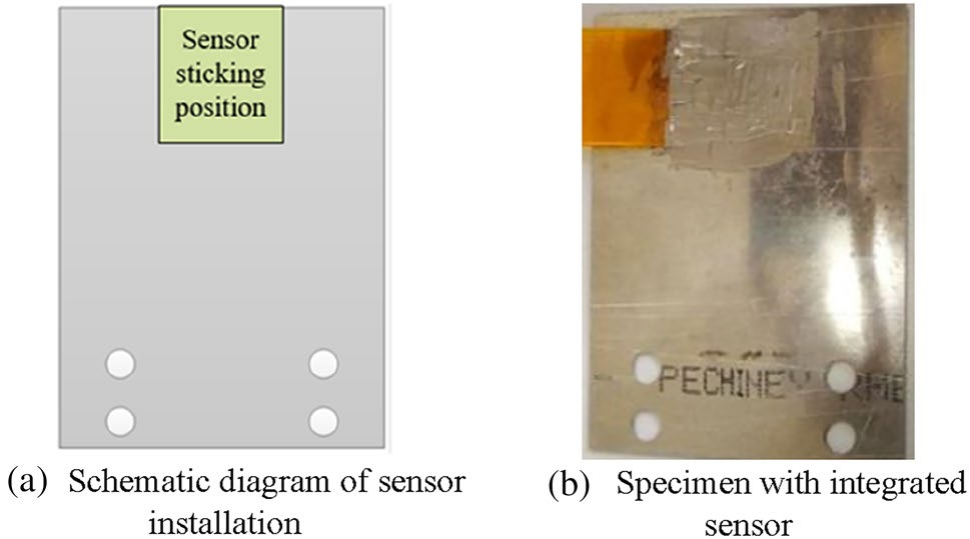
Sensor installation.

**Figure 9:**
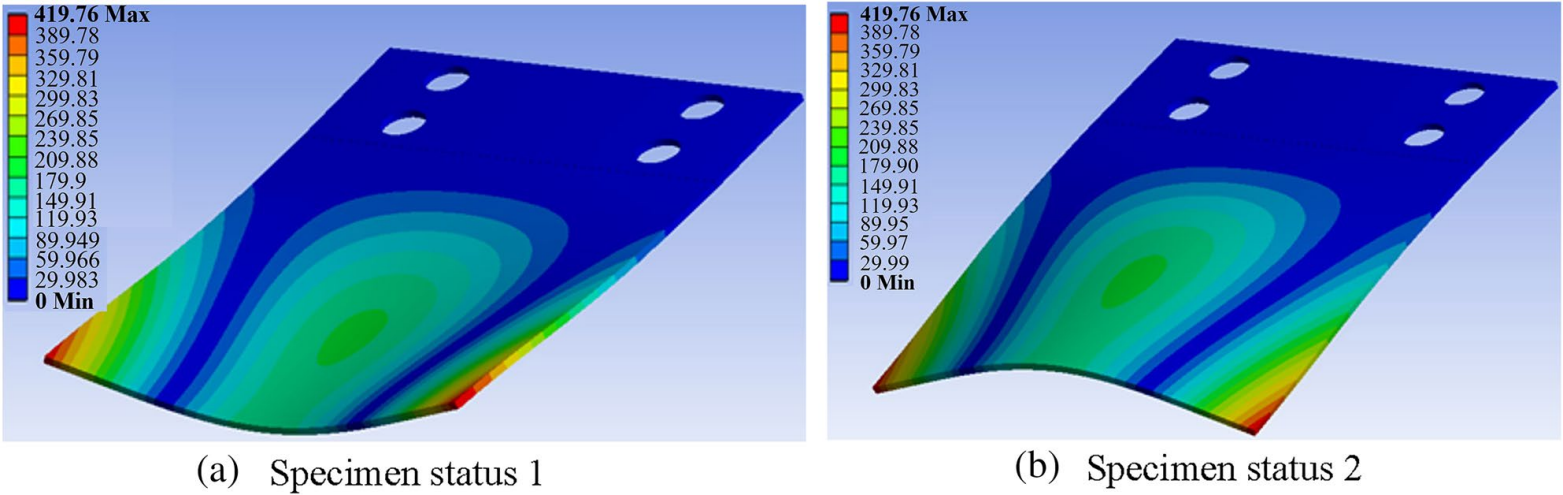
Deformation of the specimen in the fourth order vibration mode.

During the experiment, the monitoring area of the sensor is mounted at the middle of the free end of the specimen, as shown in Fig. 8. In the fourth order modal vibration, this area is the stress concentration area with the largest deformation in the specimen. In a cycle, the two maximum deformation states of the specimen are shown in Fig. 9. During the vibration, this area is in a state of reciprocating bending, and the frequency reaches 1005 times a second. Under such a large deformation and high frequency change state, the adhesion between the sensor and the specimen is very likely to fail. Therefore, in this case, the evaluation of the durability of the sensor is representative, which can meet the durability requirements of all aircraft structures.

### Experimental system

**Figure 10:**
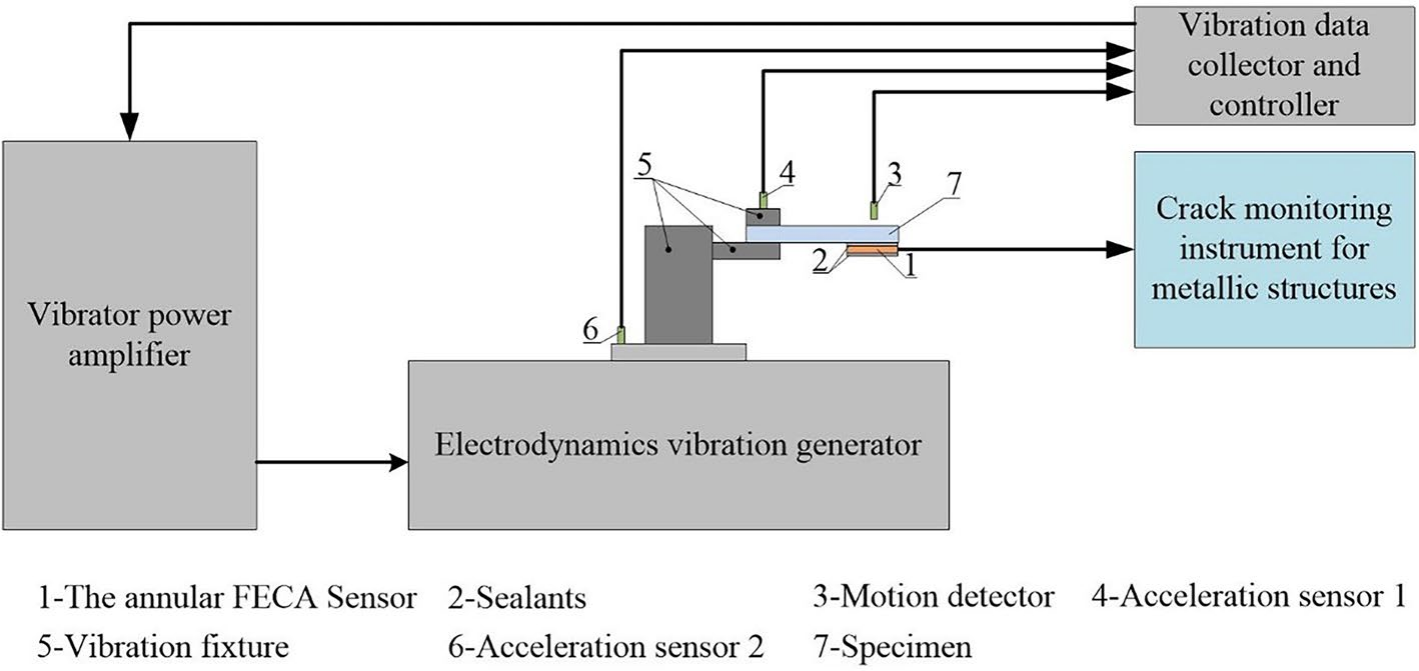
The experimental system of the durability research under vibration environment.

As illustrated in Fig. 10, the experimental system is established, so as to investigate the effect of the vibration environment on the durability of the integrated sensor. The experimental system consists of ES-50-445 vibration testing system, specimen integrated with the FECA sensor, and the crack monitoring instrument. The specimen integrated with FECA sensor is mounted on the vibration fixture, and the displacement sensor is installed directly opposite the FECA sensor to measure the vibration amplitude of the specimen. The vibration testing system controls the vibration platform by collecting the signals from the acceleration sensor, so that the vibration can output stably. During the experiment, signals of the FECA sensor are collected by the crack monitoring instrument.

### Determination of experimental vibration frequency

**Figure 11:**
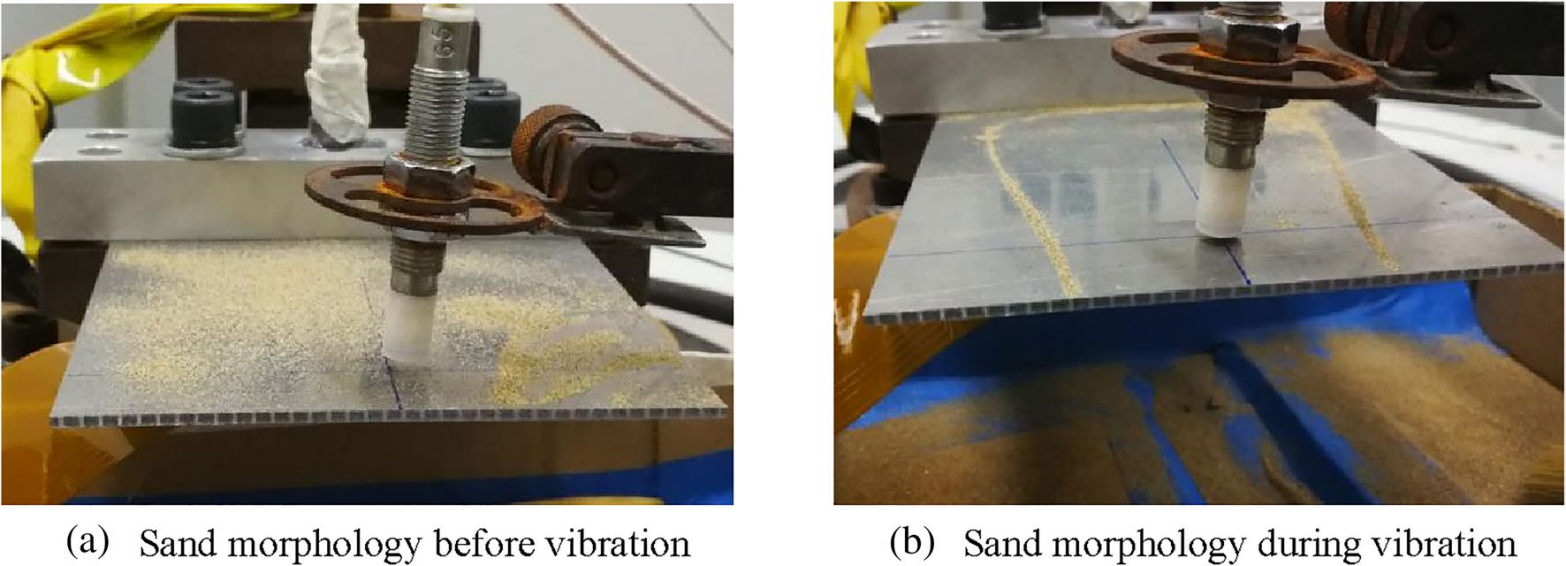
Determination of vibration mode by sand.

There must be a certain difference between the resonance frequency of the actual structure and that obtained by finite element analyses. Therefore, a frequency sweep is performed to determine the frequency of the experiment before the formal experiment. The frequency sweep is completed at an excitation magnitude of 0.8 g, and the resonance frequency of the specimen in the fourth order vibration mode is 1055 Hz. As shown in Fig. 9, the blue curved band region with zero displacement of the specimen in the fourth-order mode always exists during the experiments. Using this characteristic, this paper uses the sand to verify whether the resonance frequency obtained by the frequency sweep is the resonance frequency of the fourth-order mode. Before the vibration starts, some sand are scattered on the upper surface of the specimen, as shown in Fig. 11a. Subsequently, the experiment is performed at a vibration frequency of 1055 Hz and an excitation magnitude of 0.8 g. After the vibration started, the sand suddenly moved toward the area where the displacement of the specimen is zero, forming a sand band as shown in Fig. 11b. The morphology of the sand band is exactly the morphology of the blue region with zero displacement in the fourth-order vibration mode, which verifies that the frequency used in the experiment is the resonance frequency of the fourth order mode of the specimen.

During the formal experiment, the vibration frequency is set to 1055 Hz, and excitation magnitude gradually increases from 0.8 to 8 g. At this moment, the cycle counting start and signals from the FECA sensor are collected. During the experiment, the vibration amplitude is always stable at 0.85 mm, and the experiment stops after 10^7^ cycles.

### Experimental results

**Figure 12:**
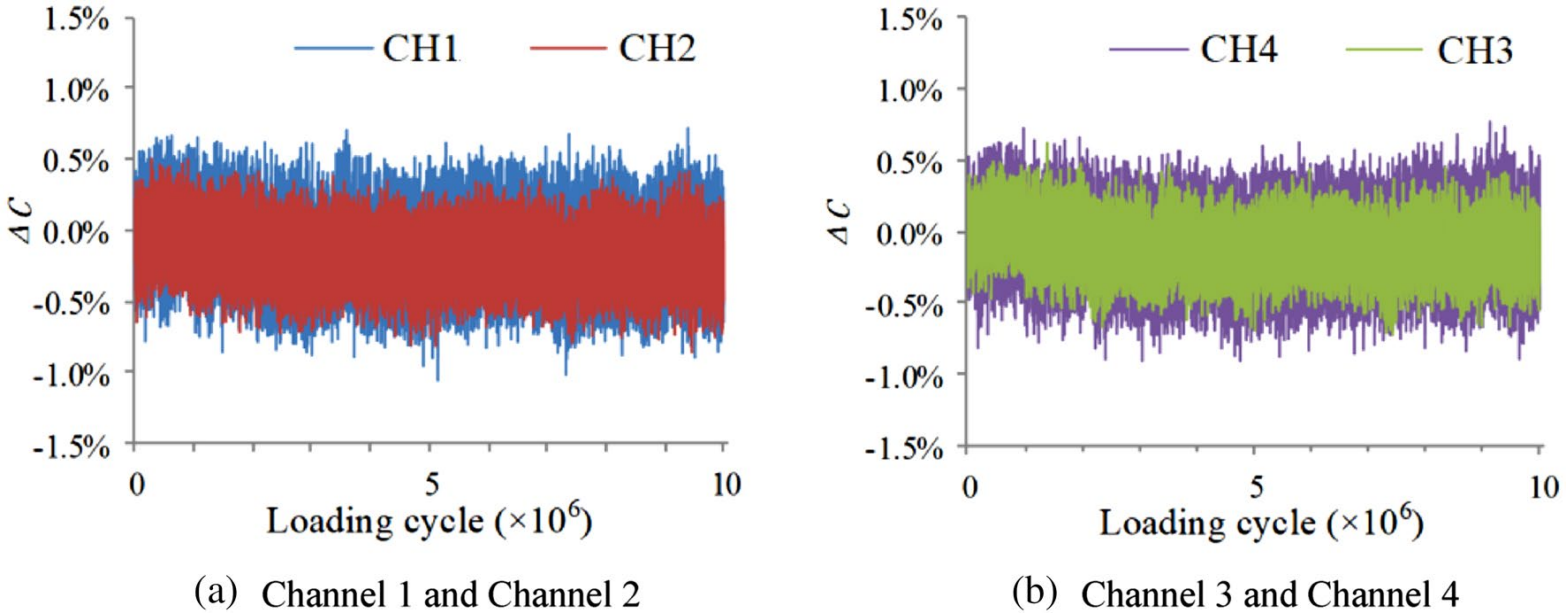
Output signals of the FECA sensor under vibration environment.

As shown in Fig. 12, output signals of the sensor keep stable, and the variation amplitudes of output signals are within 1%. After the experiment, it is found that the specimen has no crack, and the adhesion relation between the sealant and the specimen has not changed. It shows that the integrated sensor has high durability in vibration environment and can effectively resist the impact of the vibration environment.

## The durability experiments in three exposed environments

During the use of the aircraft structure, sensors are exposed to a variety of exposure environments, which will adversely affect the normal operation of FECA sensors, such as sealant aging and failure, metal corrosion, insulation failure, etc. Therefore, in this section, durability experiments in three exposed environments, including salt fog corrosion environment, fluid immersion environment, as well as hygrothermal and ultraviolet-radiation environment, are performed to evaluate the durability of FECA sensors in these three exposed environments.

### Salt fog corrosion environment experiment

**Figure 13:**
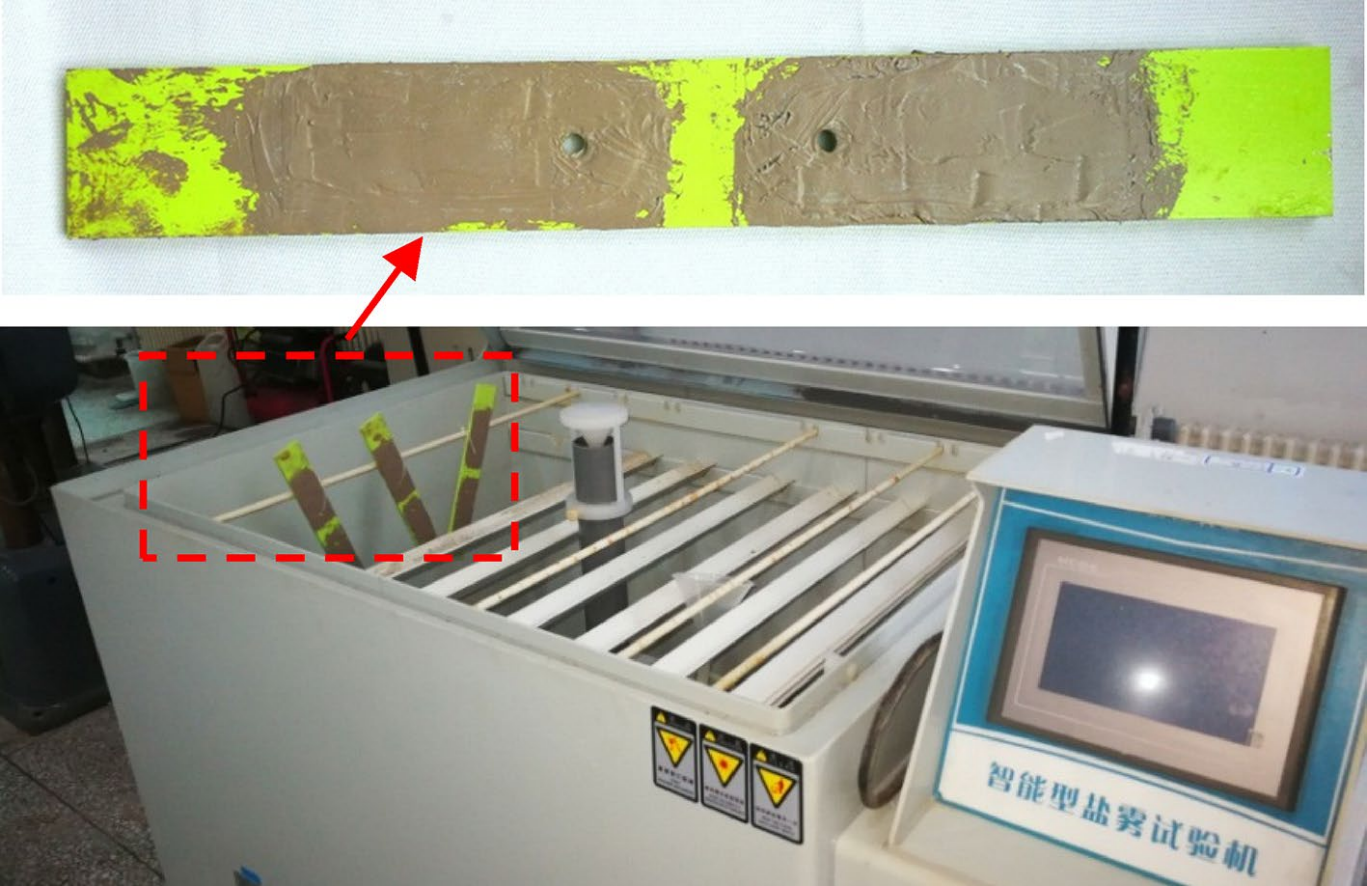
Salt fog corrosion experiment.

As shown in Fig. 13, the specimen is a 2024-T351 aluminum alloy two-hole specimen, which has a complete protective coating. Two FECA sensors are integrated on the same specimen. To verify that the sensor is working properly, the output signal of FECA sensor is collected for 50 times before experiment. According to GB/T 10125-2012^29^, the specimens are suspended in the experimental box, and the specimens are not in contact with each other. The composition and concentration of the salt fog corrosion solution are shown in Table 2. The solution is an accelerated experimental solution based on the environmental data of a place near the coast of China in the last ten years. During the experiment, the temperature in the experimental box is maintained at 38 ± 2 °C, and the salt fog sedimentation speed is measured by every 24 h to ensure that the sedimentation speed is 1–3 ml/h·80 cm^2^.The experiment lasts for 600 h.

**Table 2:** Solution concentration for accelerated salt fog corrosion experiment.

Corrosive solution	Atmospheric concentration	Accelerated experimental solution concentration (100 times)
H_2_SO_4_	0.09337	9.337
HNO_3_	7.16 × 10^–5^	7.16 × 10^–3^
NaCl	2.27	227

**Figure 14:**
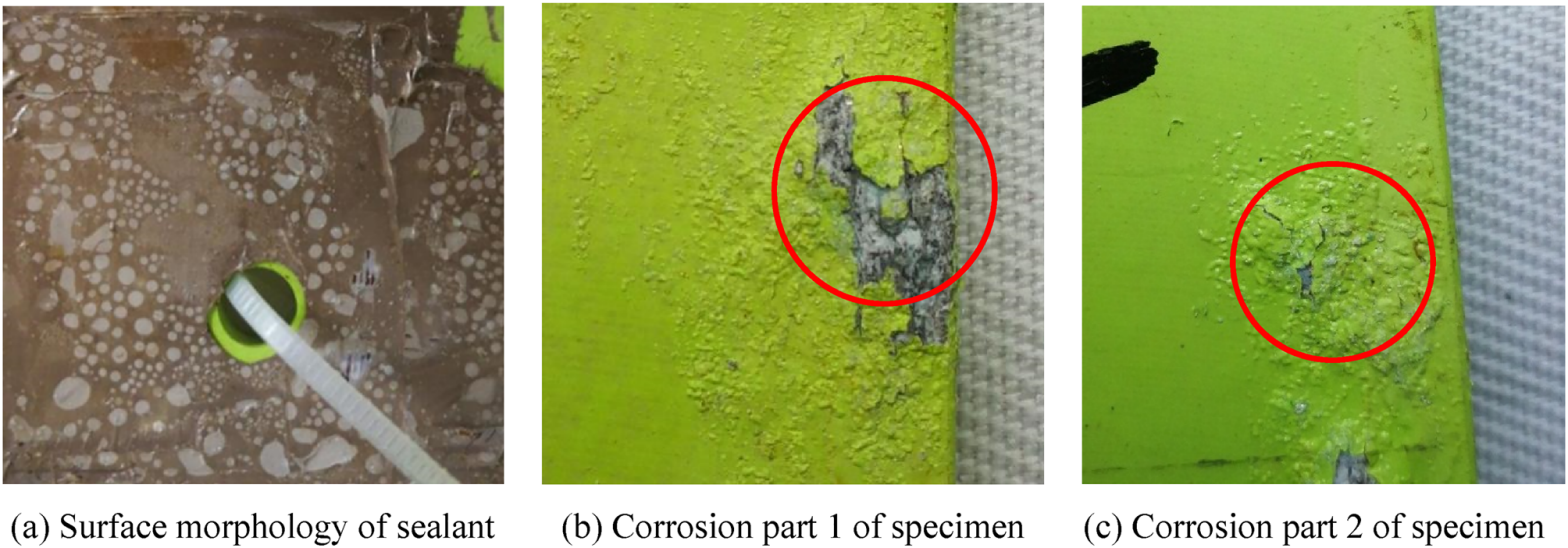
Morphology of specimen after salt fog corrosion experiment.

After the experiment, as shown in Fig. 14a, the surface of the sealant of the specimen is distributed with many white salts. The sealant is closely and completely combined with the specimen without degumming. However, two parts of the protective coatings on the specimen have failed, resulting in corrosion of the specimen substrate, as shown in Fig. 14b,c. By scraping the sealant at the sensor joint and measuring the electrical resistance of each coil, it is found that the resistance of each coil of the sensor is normal. Then, the output signal of FECA sensor is collected for 50 times. As shown in Table 3, the average value of the sensor output signals collected before and after the experiment can show that the sensor is in good condition. The experimental results show that, even if the protective coating of the structure is partially corroded, the integrated sensor has good durability in salt fog corrosion environment.

**Table 3:** The average value of the sensor output signal.

Group	CH1 (%)	CH2 (%)	CH3 (%)	CH4 (%)
Before experiment	0.2	0.5	0.3	0.5
After experiment	0.7	0.4	0.3	0.8

**Figure 15:**
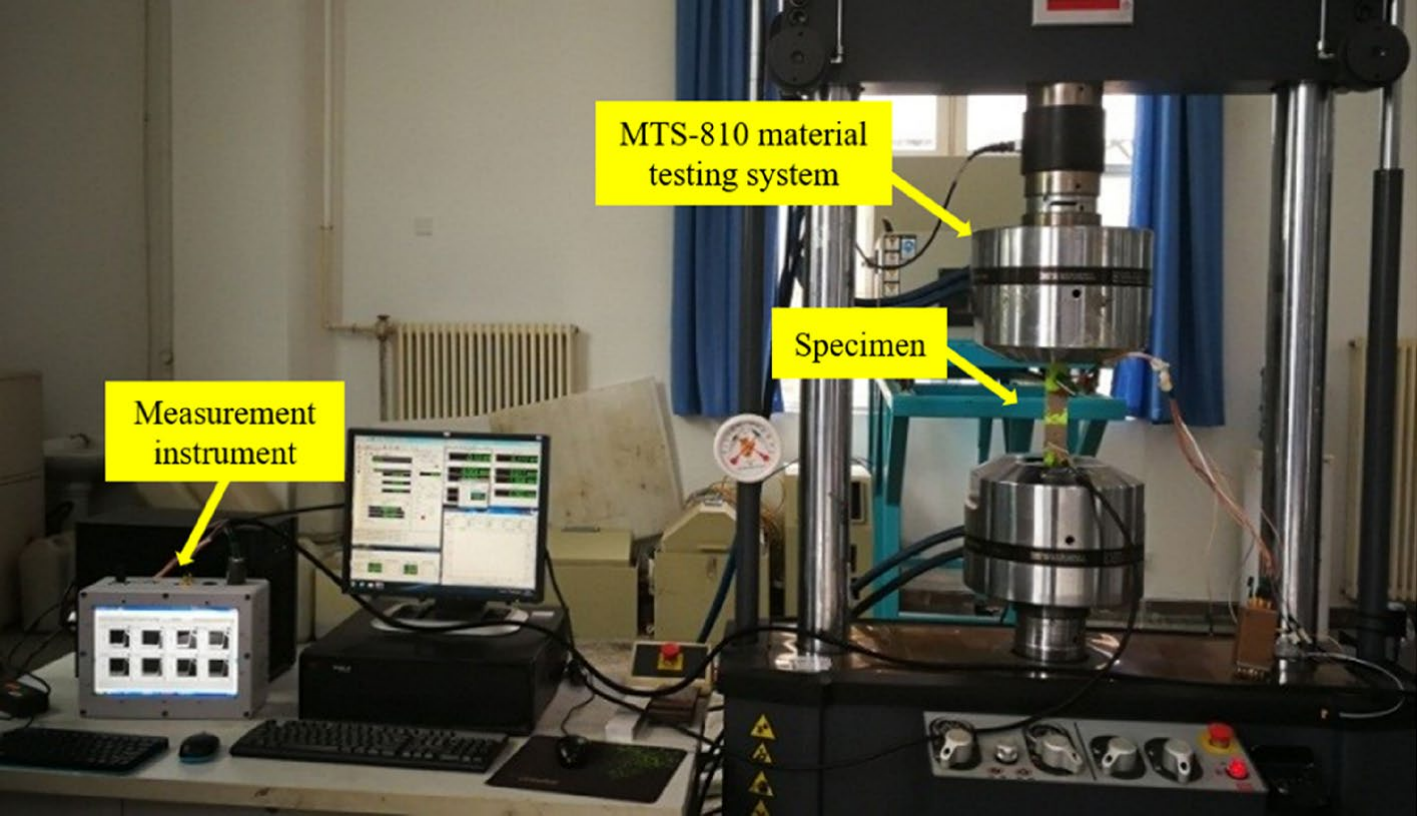
Structural fatigue crack monitoring experiment.

The fatigue experiment under constant amplitude spectrum is carried out on the specimen after salt fog corrosion experiment, and its parameters are shown in Table 4. As shown in Fig. 15, the experimental system includes a measurement instrument, MTS810 material testing system and specimens integrated with FECA sensors.

**Table 4:** Constant amplitude spectrum parameter.

Parameter	Maximum stress	Stress ratio	Loading frequency
Number	145 MPa	0.06	15 Hz

**Figure 16:**
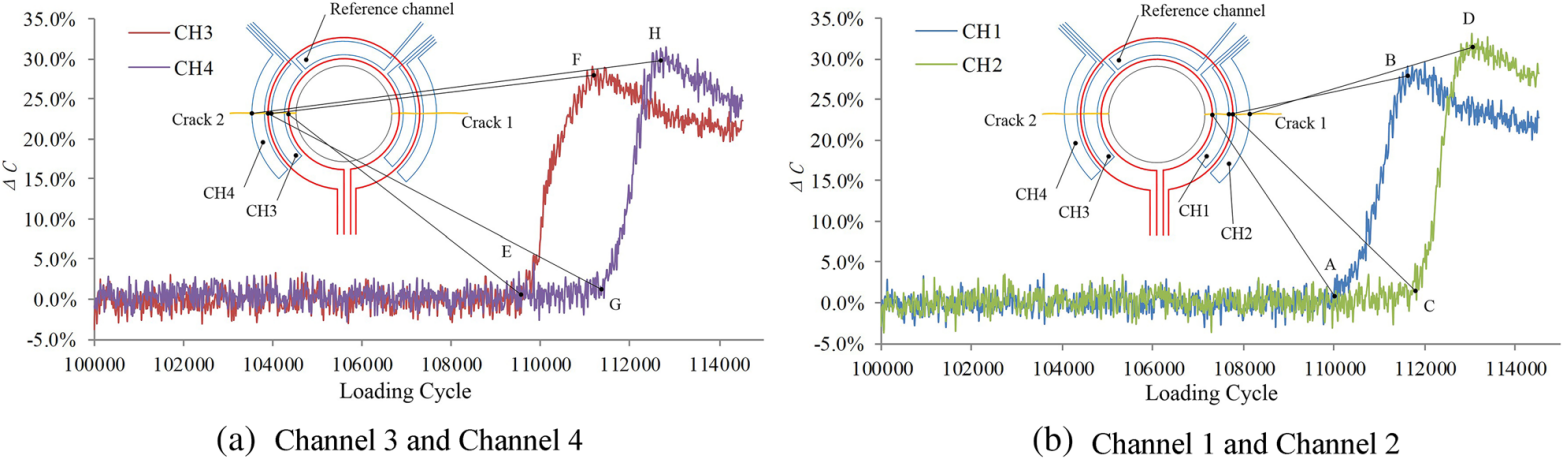
Relationship between sensor output signal and the number of loading cycles.

After 114,526 fatigue cycles, the bolt hole in the upper part of the specimen is fractured. As shown in Fig. 16, the output signal of channel 3 starts to increase when the loading cycle reaches 109,618 cycles, and the crack length on the left side of the structure is 1 mm. When the loading cycle reaches 111,144 cycles, the output signal of channel 3 reaches the maximum inflection point, indicating that the crack length side is 2 mm.When the loading cycle reaches 111,340 cycles, the output signal of channel 4 starts to increase, indicating that the crack length side is 2.2 mm.When the loading cycle reaches 112,593 cycles, the output signal of channel 4 reaches the maximum inflection point, indicating that the crack length side is 3.2 mm.The output signals of channel 1 and channel 2 of the sensor reach the inflection points A, B, C and D when the number of loading cycle is 110,042, 111,536, 111,778 and 112,865. The corresponding crack length on the right side is 1 mm, 2 mm, 2.2 mm and 3.2 mm, respectively.

The experimental results show that even if the concentration of the corrosive medium is 100 times the environmental monitoring value, the bond between the sensor and the structure is not affected by the salt fog corrosion environment, and the integrated sensor can effectively resist the influence of the salt fog corrosion environment. The integrated FECA sensor can still effectively monitor structural cracks.

### Fluid immersion environmental experiment

**Figure 17:**
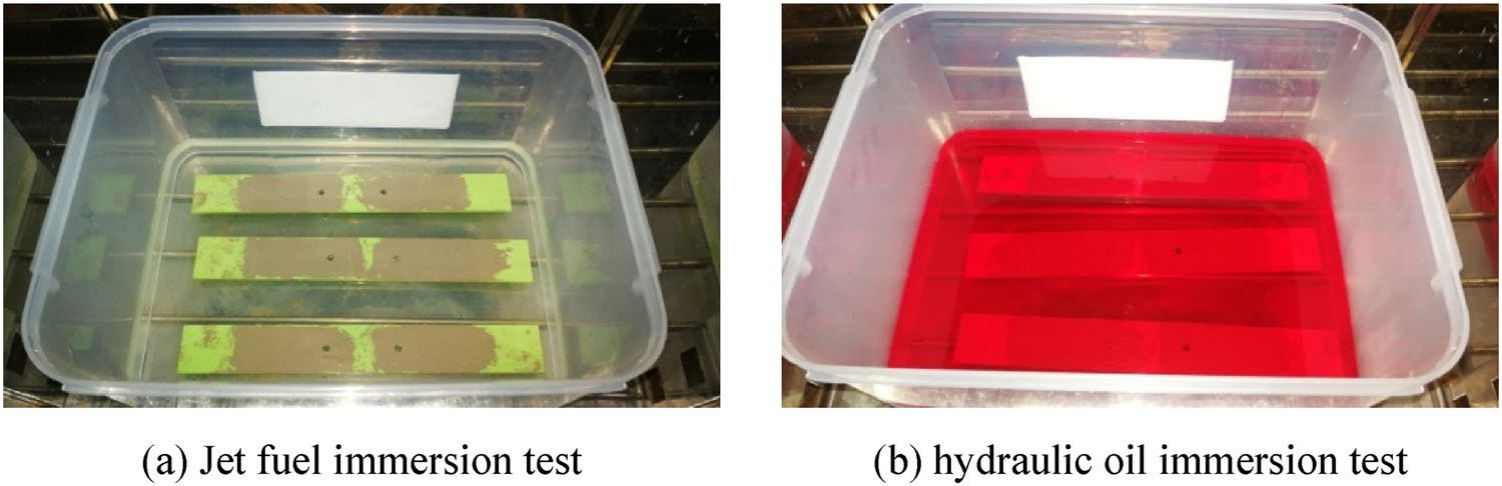
Fluid immersion experiment.

According to the American standard RTCA DO-160G^30^, jet fuel and hydraulic oil immersion experiments are completed. Before the experiments, the output signal of FECA sensor is collected for 50 times. Then, the jet fuel immersion experiment is performed first, and the RP-5 jet fuel is selected for the experiment. The fuel has a high flash point higher than 60 °C, which is mainly used for land-based and carrier-based aircraft equipped with gas turbine engines. As shown in Fig. 17a, the specimen is completely immersed in the RP-5 jet fuel, and the container is placed in an environmental chamber. The temperature of the environmental chamber is maintained at 45 °C and the experiment lasts for 800 h.

The specimen soaked with jet fuel is used for hydraulic oil immersion experiment after drying, and No. 15 aviation hydraulic oil is used for experiment. No. 15 aviation hydraulic oil is a petroleum-based hydraulic oil used in the main and auxiliary hydraulic systems of aircrafts. The flash point of this hydraulic oil is higher than 82 °C. As shown in Fig. 17b, the specimen is completely immersed in hydraulic oil, and the container is placed in an environmental chamber for 500 h, whose temperature is maintained at 70 °C.

**Figure 18:**
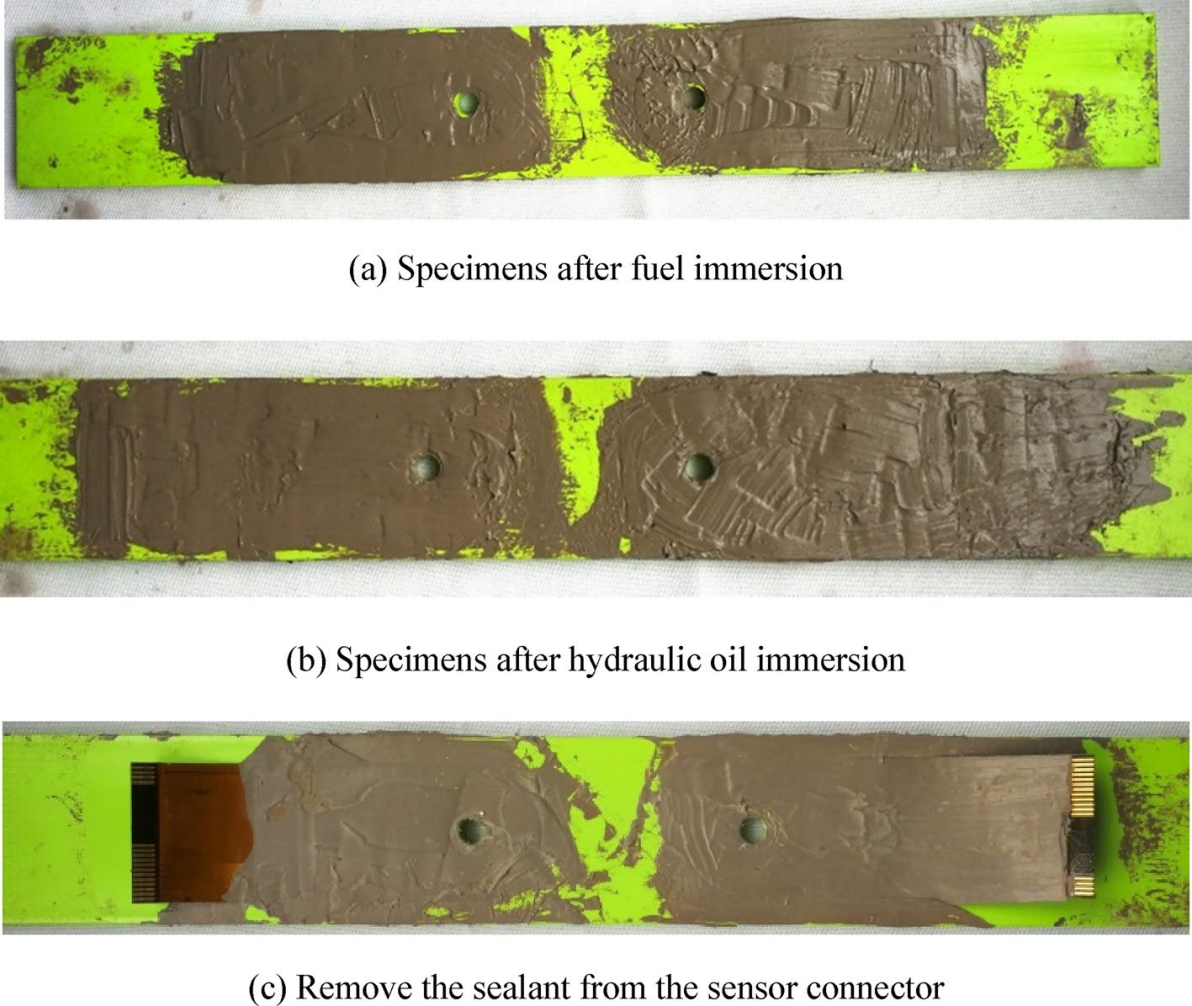
Specimen after fluid immersion experiment.

The morphology of the specimen after the fluid immersion environmental experiment is shown in Fig. 18a,b, and it is found that the sealant is intact and adhered tightly, and no debonding is found. Then, as shown in Fig. 18c, the sealant of the sensor joint part is scraped off to measure the resistance of each coil, and it is found that the resistance values of the resistance of each coil are normal, indicating that the sensor has not failed. Then, the output signal of FECA sensor is collected for 50 times. As shown in Table 5, the average value of the sensor output signals collected before and after the experiments show that the sensor is in good condition and the fluid immersion doesn’t have a harmful effect on integrated sensors.

**Table 5:** The average value of the sensor output signal.

Group	CH1 (%)	CH2 (%)	CH3 (%)	CH4 (%)
Before experiments	0.6	0.2	0.3	0.1
After experiments	0.4	0.2	0.4	0.1

**Figure 19:**
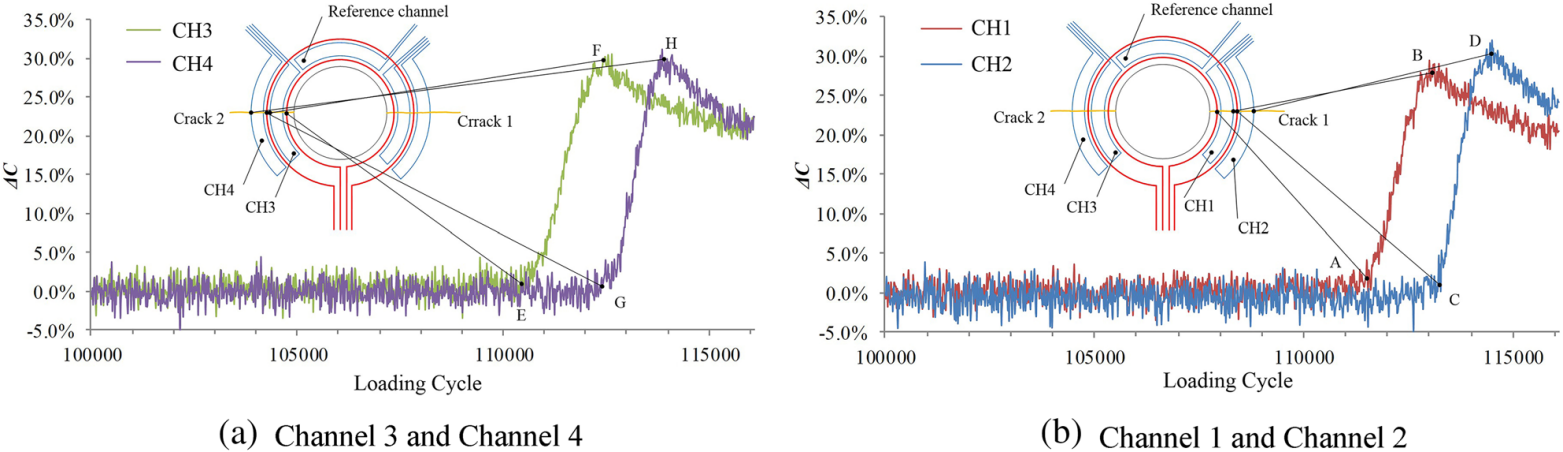
Relationship between sensor output signal and number of load cycles.

According to the parameters in Table 4, the specimen after the liquid immersion experiment is subjected to a fatigue crack monitoring experiment. After 116,097 fatigue cycles, the bolt hole in the upper part of the specimen is fractured. The output signal of the sensor is shown in Fig. 19, and the monitoring results are shown in Table 6.

**Table 6:** Monitoring results in fluid immersion environmental experiment.

Channel	CH1		CH2		CH3		CH4	
Inflection point	A	B	C	D	E	F	G	H
Crack lengths	1	2	2.2	3.2	1	2	2.2	3.2
Loading cycle	111,521	112,926	113,152	114,315	110,313	112,246	112,578	113,771

The experimental results show that the integrated sensor can withstand the harmful effects of jet fuel and hydraulic oil, the adhesion between the sensor and the structure will not be affected by the immersion liquid, and the integrated sensor can still effectively monitor the structure crack.

### Hygrothermal and ultraviolet radiation environmental experiment

**Figure 20:**
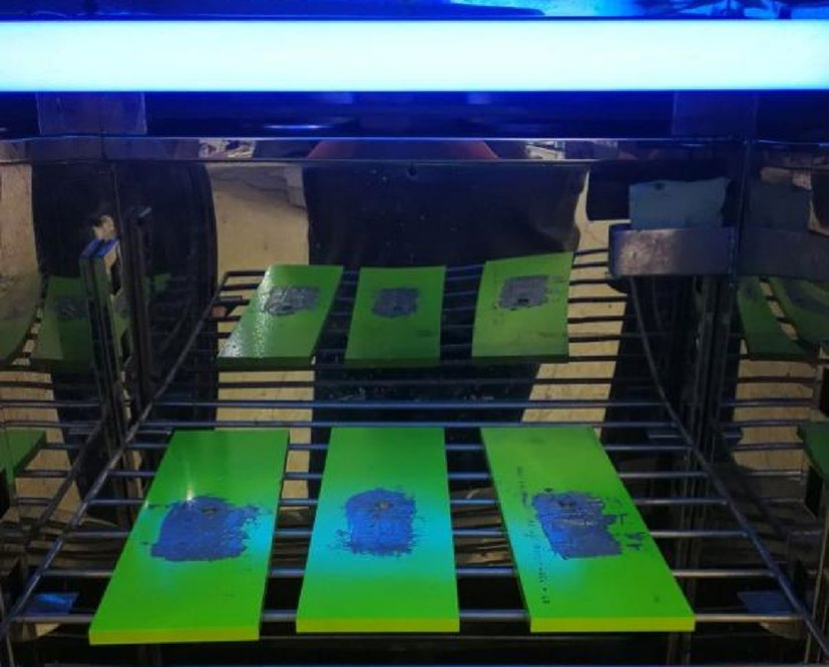
Hygrothermal and UV radiation environmental experiment.

As shown in Fig. 20, specimens integrating sensors are placed in an environmental chamber to carry out a hygrothermal experiment and an ultraviolet radiation experiment at the same time^31^. And, the output signal of FECA sensor is collected for 50 times before the experiment. The experiment is performed for 600 h. During the experiment, the ambient temperature is maintained at 80 °C, the relative humidity is 95%, the ultraviolet wavelength is 340 nm, and the intensity of ultraviolet radiation is 70 W/m^2^. Moreover, distilled water is used in the experiment, and the supplementary experiment water is checked in due course.

**Figure 21:**
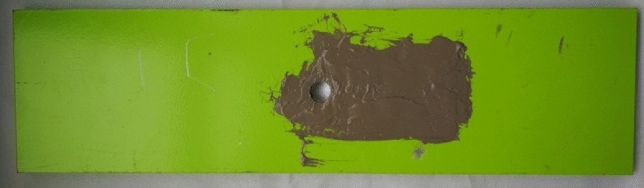
Surface of the specimen after Hygrothermal and ultraviolet radiation environmental experiment.

After the experiment, the specimens are dried, and the shape of the specimens are shown in Fig. 21. The inspection found that the sealant is intact and the bonds between sensor and the specimen are still tight. After scraping off the joints of the sensor and measuring the resistance of each coil, it is found that the resistance of each coil is normal. Then, the output signal of FECA sensor is collected for 50 times. As shown in Table 7, the average value of the sensor output signals collected before and after the experiment can indicate that the sensor is in good condition.

**Table 7:** The average value of the sensor output signal.

Group	CH1 (%)	CH2 (%)	CH3 (%)	CH4 (%)
Before experiment	0.7	0.5	0.2	0.1
After experiment	1.0	0.4	0.3	0.1

**Figure 22:**
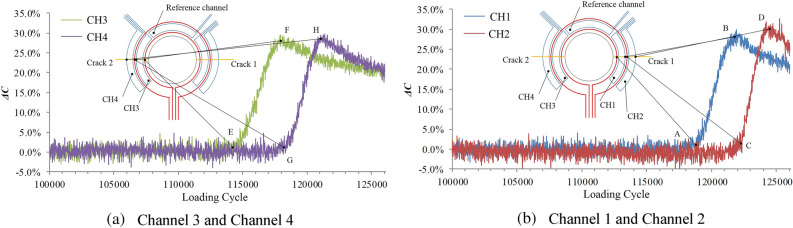
Relationship between sensor output signal and number of load cycles.

Fatigue crack monitoring experiments are performed on the specimens after the humid-thermal and ultraviolet radiation experiment according to the method described in “Salt fog corrosion environment experiment” section. After 126,032 fatigue cycles, the specimen is fractured from the edge of the hole. The output signal of the sensor is shown in Fig. 22, and monitoring results are shown in Table 8.

**Table 8:** Monitoring results in hygrothermal and ultraviolet radiation environmental experiment.

Channel	CH1		CH2		CH3		CH4	
Inflection point	A	B	C	D	E	F	G	H
Crack lengths	1	2	2.2	3.2	1	2	2.2	3.2
Loading cycle	118,845	121,548	122,182	124,145	114,617	117,712	118,241	120,944

The experimental results show that the hygrothermal and ultraviolet radiation environment will not have harmful effects on integrated sensors, and the adhesion between the sensor and the structure will not be affected. Sensors can still effectively monitor the structure crack.

## Conclusions

During the engineering use of the FECA sensor, the service environment has a great influence on sensors. In this paper, FECA sensors are taken as the research object, and one integrated method of the sensor is studied. In order to study the durability of sensors in harsh service environments on this basis, the durability experiments in vibration environment and exposure environments are carried out.

1. The surfaces of FECA sensors are covered with PI protective layer, which can provide a certain protection for the sensor.
2. According to the finite element simulation results, the fourth order vibration mode is selected to carry out the durability experiment in vibration environment. The experimental results show that the output signal of the sensor is stable and the amplitude of the output signal variation is within 1% after 10^7^ cycles at a vibration frequency of 1055 Hz, which means the integrated sensor has high durability in vibration environment.
3. Three harsh service exposed environments are selected for durability experiments respectively. The experimental results show that integrated sensors have high durability in salt fog corrosion environment, fluid immersion environment, as well as hygrothermal and ultraviolet radiation environment. In these environments, sensors can effectively monitor structural cracks.

In summary, FECA sensors integrated by aviation sealants have high durability in harsh service environments of aircraft. The research results can provide important support for the engineering applications of FECA sensors.
